# A Pilot Study of Chronological Microbiota Changes in a Rat Apical Periodontitis Model

**DOI:** 10.3390/microorganisms8081174

**Published:** 2020-08-02

**Authors:** Ok-Jin Park, Moon-Hee Jeong, Eun-Hye Lee, Mi-Ran Cho, Jaehong Hwang, Seungryong Cho, Cheol-Heui Yun, Seung Hyun Han, Sun-Young Kim

**Affiliations:** 1 Department of Oral Microbiology and Immunology, DRI, and BK21 Plus Program, School of Dentistry, Seoul National University, Seoul 08826, Korea; okjpark7@snu.ac.kr; 2 Department of Conservative Dentistry and Dental Research Institute, School of Dentistry, Seoul National University, Seoul 03080, Korea; connie1028@naver.com (M.-H.J.); eunhye789@hanmail.net (E.-H.L.); rudy311@naver.com (M.-R.C.); 3 Department of Nuclear and Quantum Engineering, KAIST, Daejeon 34141, Korea; jhhwang6@kaist.ac.kr (J.H.); scho@kaist.ac.kr (S.C.); 4 Department of Agricultural Biotechnology and Research Institute for Agriculture and Life Sciences, Seoul National University, Seoul 08826, Korea; cyun@snu.ac.kr

**Keywords:** apical periodontitis, Rat apical periodontitis model, Microbiota, In vivo micro-computed tomography

## Abstract

Apical periodontitis caused by microbial infection in the dental pulp is characterized by inflammation, destruction of the pulpal and periradicular tissues, and alveolar bone resorption. We analyzed the chronological changes in microbiota using a pyrosequencing-based approach combined with radiologic and histopathologic changes in a rat apical periodontitis model. During the three-week observation, the pulp and periapical area showed a typical progress of apical periodontitis. A total of 27 phyla, 645 genera, and 1276 species were identified. The root apex had a lower bacterial species diversity than the pulp chamber. Proteobacteria, Firmicutes, Bacteroidetes, and Actinobacteria were dominant phyla in both the pulp chamber and root apex. Remarkably, bacterial communities showed a tendency to change in the root apex based on the disease progression. At the genus level, *Escherichia, Streptococcus, Lactobacillus, Rodentibacter*, and *Bacteroidetes* were dominant genera in the pulp chamber. The most abundant genera in the root apex were *Bradyrhizobium, Halomonas*, and *Escherichia*. The species *Azospirillum oryzae* increased in the pulp chamber, whereas the species *Bradyrhizobium japonicum* and *Halomonas stevensii* were highly observed in the root apex as the disease progressed. The experimental rat model of apical periodontitis demonstrated a relationship between the microbiota and the apical periodontitis progression.

## 1. Introduction

Apical periodontitis is an inflammatory disease of the periradicular tissues caused by bacterial infections in the dental pulp system, mostly due to caries, defective restorations, trauma, or failure of root canal treatment [1,2]. The invading bacteria and their by-products activate various immune cells or nonimmune cells that cause acute or chronic inflammatory responses in the dental pulp and the surrounding area of the root apex, consequently resulting in pulpal destruction and induction of a periapical lesion with alveolar bone resorption [3].

The relationship between the development of apical periodontitis and the role of bacteria has been a major research theme for many years. Previous studies on endodontic infection using an anaerobic culture method attempted to reveal several bacteria as the main causative pathogen, thus concluding that Gram-negative and anaerobic bacteria are predominant in endodontic infection [4,5,6]. However, a later study using a molecular approach demonstrated that apical periodontitis is caused by a mixed infection of endogenous bacteria [7]. Recently, the development of sequencing tools has resulted in increased insight into the bacterial diversity of the infected root canal system. Next-generation sequencing using a pyrosequencing method has efficiently and accurately identified even a minor population of bacterial species, thereby offering deep insight into apical periodontitis [8,9]. Most pyrosequencing studies for apical periodontitis have collected human endodontic infection samples and compared high-throughput microbiota under variable conditions, such as acute versus chronic [10], primary versus persistent infections [11], and symptomatic versus asymptomatic infections [12].

Microbial communities can alter their profiles to adapt to environmental conditions for survival and maintenance [13]. Likewise, the microbiota in the dental pulp system, such as the pulp chamber, root canal, and apical area, are presumed to alter their profiles as apical periodontitis develops, but details on this microbiota change are poorly understood in both humans and animals [9]. Thus far, studies on the endodontic microbiota in humans have used mostly the serum from the root canal or a part of an extracted tooth and have shown a bacterial community profile only at a specific stage of the disease, not the microbiota shift during the progress of apical periodontitis [8,11,12]. Comprehensive approaches to the chronological changes in the microbiota of the root canal using radiologic and histopathologic analyses are viable only in animal studies because of ethical issues. Although bacterial diversity in the root canal during the expansion of the periapical lesion of rats was shown in a previous study [14], this study used an outdated culture method and did not apply state-of-the-art techniques and conduct a comprehensive investigation of periapical lesion development. There is still room for a comprehensive study on the alteration of microbiota together with histopathologic changes using the progression of apical periodontitis.

The present study mainly aimed to investigate the chronological changes in the endodontic microbiota in a rat model with progression in apical periodontitis. We used in vivo micro-computed tomography (micro-CT), histomorphometry, and pyrosequencing in a rat experimental model of apical periodontitis to obtain a comprehensive understanding of the change of microbial diversity.

## 2. Materials and Methods

### 2.1. Animals

**Figure 1:**
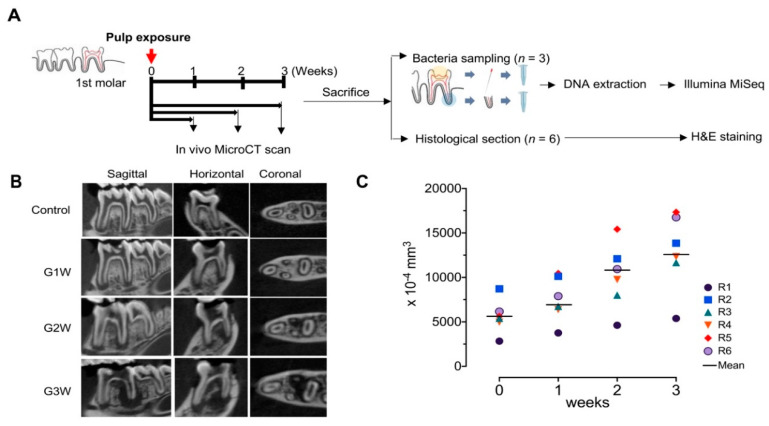
(**A**) Scheme of the animal experiment in this study. (**B**) Representative in vivo micro-computed tomography (micro-CT) images for three different axes for the chronological changes of the pulp-exposed lower first molar of rat. (**C**) Change in the volume of the periradicular radiolucent area for each rat of the G3W group during the three-week observation.

The animals used were six-week-old female pathogen-free Sprague Dawley rats (weight: 180–220 g; Orient Bio, Seongnam, Korea). The Institutional Animal Care and Use Committee of Seoul National University approved the present study (authorization no. SNU-180607-2). Figure 1A presents the overall experimental scheme for the rats.

### 2.2. Pulp Exposure

The rats were randomly divided into a control group with no pulp exposure (n = 2) and three experimental groups (n = 6)—G1W, animals with one week of pulp exposure; G2W, animals with two weeks of pulp exposure; and G3W, animals with three weeks of pulp exposure. The rats were anesthetized using an intraperitoneal injection of mixtures of Zoletil (Virbac, Carros, France; 0.05 mL/100 g) and Rompun (Bayer, Leverkusen, Germany; 0.03 mL/100 g). The field of operation was disinfected with 10% povidone–iodine. The cavity for access was prepared on the bilateral first mandibular molars to expose the pulp using the # 1/4 dental round bur in high-speed rotation under a surgical microscope (Carl Zeiss Meditec AG, Jena, Germany). Teeth were left open to the oral environment for 1, 2, or 3 week(s) for each group after plugging the exposed pulp chamber with a sterile cotton pellet.

### 2.3. In Vivo Micro-CT Imaging and Analysis

For the rats in the G3W group, in vivo micro-CT was conducted every week for three weeks after pulp exposure. The rats were anesthetized using the same concentrations of mixtures of Zoletil and Rompen as pulp exposure. Anesthesia was maintained by 1% isoflurane inhalation at a constant flow of 1.0 L/min. The animals were placed in a micro-CT test bed (Quantum GX, Perkin Elmer Inc., Akron, OH, USA) with the head fixed by a strap. The scanning conditions were 90 kVp, 88 uA, and a scan duration of 4.5 min. Regions of interests (ROIs) around the lower molar area were defined from the original head scan through subvolume reconstruction to create high-resolution images (30 μm pixel size). Three-dimensional (3D) reconstruction and analysis of the periapical lesion volume were conducted using data from the in vivo micro-CT of 1, 2, and 3 week(s) for only the G3W animals to track the periapical change for the same tooth. We aligned the micro-CT volume images using a mutual information-based 3D registration technique with the reference images acquired initially, to ensure a consistent analysis of the lesion development over time. Mutual information-based image registration techniques are widely used in the medical imaging field and are particularly suitable for the present study’s alignment purpose, considering the relatively high contrast of the micro-CT image [15]. In the aligned ROI images, the lesions were segmented automatically using the k-means clustering method and the region growing method in tandem [16,17]. The lesion volume was then calculated for each segmented lesion.

### 2.4. Histopathologic Analysis

After sacrifice, half of the mandibles were fixed with 4% paraformaldehyde for 12 h and decalcified with buffered (pH 8.0) 17% EDTA solution, which was exchanged every other day for five weeks with gentle stirring at room temperature. After dehydration with serial concentrations of ethanol (70–100%), the samples were embedded in paraffin. The samples were serially sectioned (4 μm), stained with hematoxylin–eosin (H&E), and examined with an optical microscope (DM 5000 B, Leica, Wetzlar, Germany) as previously described [18]. The pulp necrosis level was assessed histometrically for the distal root canal of each tooth (n = 6). The pulp necrosis level was labeled as follows: 0 (pulp cells are alive in the whole root), 1 (partial necrosis occurs in the coronal one-third of the root canal), 2 (necrosis occurs in two-thirds of the whole root canal), and 3 (full necrosis of the whole root canal). Morphometric evaluation of the periapical lesion size was also conducted for the distal root and surrounding area with the slides containing the open apical foramen of the distal root. Similar to a previous study [19], the H&E-stained slides were observed in fluorescent mode using a bandpass filter (excitation filter: BP 480/40 nm, dichromatic mirror: 505 nm, suppression filter: BP 527/30 nm). The size of the periapical lesion was delineated for the areas of bone resorption around the distal root. The normal width of the periodontal ligament space was measured from the control group, and the mean value was 129 μm. The lesion size was calculated using measuring software (LAS-X, Leica) by subtracting the normal periodontal ligament space from the delineation.

### 2.5. Sampling, Genomic DNA Extraction, Amplification, and Sequencing

Bacterial sample collection was conducted for the pulp chamber and periapical area. The other half of the sacrificed rat mandible was used for sample collection. Three mandibles from each observation period were randomly selected, and their soft tissue was peeled off with #15 scalpel and micro-tweezer. The first molar and surrounding area were disinfected with 10% povidone–iodine, except for the accessed cavity of the occlusal surface. To sample bacteria from the pulp chamber, the cotton pellet was removed from the pulp chamber. The pulp chamber was then filled with 1 μL of phosphate-buffered saline and agitated with a sterile #25 K-file. A sterile paper point was introduced into the bottom of the pulp chamber and then delivered to an Eppendorf tube (n = 3). To sample bacteria at the apical part, the cortical bone around the mandibular first molar was cut and peeled off using a sterilized low-speed disk and micro-tweezer. The apical one-third of the exposed mesial root was cut using a sterile #15 scalpel and transferred to an Eppendorf tube (n = 3). For bacterial sampling for saliva, a sterile paper point was introduced under the tongue of the rats in the control group that were of the same age as those in the G3W group. The wet paper point was then delivered to an Eppendorf tube (n = 2). Using a G-spin genomic DNA (gDNA) extraction kit (iNtRon Biotechnology, Gyeonggi, Korea), gDNA was extracted from the paper points and roots according to the manufacturer’s instructions. The extracted gDNA was then subjected to polymerase chain reaction (PCR) using primers targeting the V3 to V4 regions of the bacterial 16S ribosomal RNA gene. The PCR products were purified with the CleanPCR (CleanNA, Alpehn aande Rijn, The Netherlands), and the quality and product size were assessed on the Bioanalyzer 2100 (Agilent, Palo Alto, CA, USA) using a DNA 7500 chip. Mixed amplicons were pooled, and DNA sequencing was conducted at ChunLab, Inc. (Seoul, Korea) using an Illumina MiSeq sequencing system (Illumina Inc., San Diego, CA, USA) according to the manufacturer’s instructions. The raw sequence data are deposited at the NCBI Short Read Archive under BioProject ID PRJNA640939.

### 2.6. Taxonomic Assignment of Individual Sequencing Reads, Bioinformatics, and Statistical Analysis

Sequencing reads without ambiguous reads (n ≥ 2), and chimeric sequences were processed for the bioinformatics analysis as previously described [20,21]. The EzBioCloud 16S rRNA database was used for the taxonomic assignment of each sequencing read using usearch_global command of VSEARCH, followed by more precise pairwise alignment. The cutoff value for assigning a sequence to a species-level phylotype was ≥ 97% similarity. Cluster_fast command was used to perform de-novo clustering to generate additional operational taxonomic units (OTUs). Alpha diversity (Shannon index) was conducted by using CL community version 3.46 (Chunlab, Inc., Seoul, Korea). Wilcoxon rank-sum test was used to compare OTUs and Shannon index. The differences in the bacterial community patterns among the G1W, G2W, and G3W groups were evaluated using the Kruskal–Wallis test. Principal coordinate analysis was performed using the unweighted pair group method with arithmetic mean (UPGMA) with the weighted Fast UniFrac.

## 3. Results

### 3.1. In Vivo Micro-CT Observations and Analysis

Using 3D reconstruction of in vivo micro-CT data and analysis of the periapical lesion volume, we monitored the progression of apical periodontitis and investigated the subsequent bony change around the roots (Figure 1A). As shown in Figure 1B, all experimental groups developed periapical lesions with signs of apical periodontitis on the mesial, distal, buccal, and lingual root apices, whereas the control groups showed normal periodontal ligament space and alveolar bone structures. The periapical lesion size increased as time passed over three weeks (Figure 1B). The measured volume of the radiolucent area of all rats in the G3W group also increased over three weeks (Figure 1C). The average volume sums of the radiolucent area were 0.56 ± 0.19, 0.76 ± 0.25, 1.01 ± 0.37, and 1.26 ± 0.40 mm^3^ at 0, 1, 2, and 3 week(s), respectively.

### 3.2. Histologic Features of Apical Periodontitis Progression

**Figure 2:**
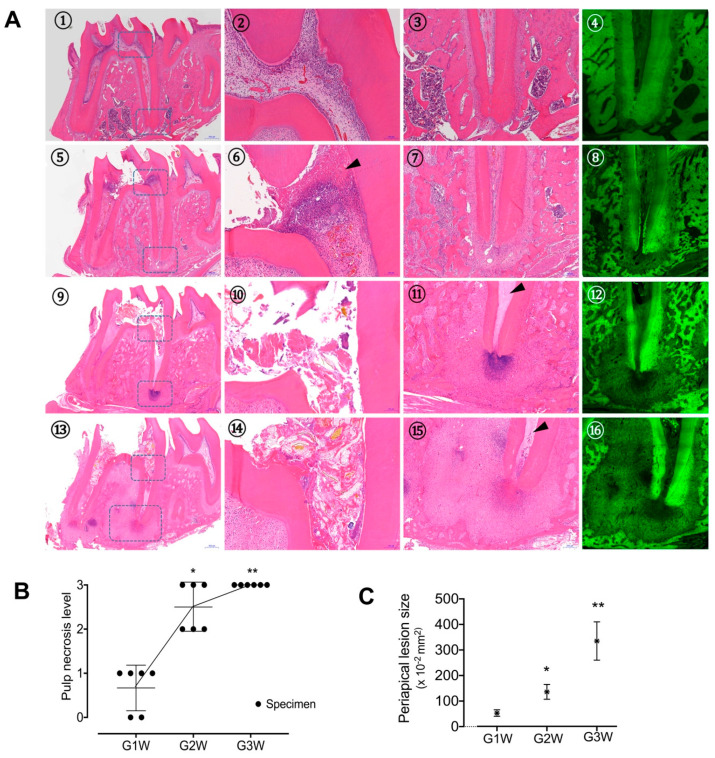
(**A**) Representative histologic features of the chronological changes in the pulp-exposed lower first molar of rat: whole tooth (1), magnified coronal pulp (2), magnified apical area (3), and fluorescence mode image of the apical area (4) for the control group without pulp exposure; whole tooth (5), magnified coronal pulp (6), magnified apical area (7), and fluorescence mode image of the apical area (8) at one week after pulp exposure; whole tooth (9), magnified coronal pulp (10), magnified apical area (11), and fluorescence mode image of apical area (12) at two weeks after pulp exposure; whole tooth (13), magnified coronal pulp (14), magnified apical area (15), and fluorescence mode image of apical area (16) at tree weeks after pulp exposure. The arrowhead indicates the pulp necrosis area. (**B**) Pulp necrosis level for the roots of all specimens. Here, 0 indicates pulp cells are alive in the whole root, 1 indicates partial necrosis has occurred in the coronal one-third of the root canal, 2 indicates necrosis has occurred in two-thirds of the whole root canal, and 3 indicates full necrosis of the whole root canal. * statistically significant difference from the G1W group (*P* < 0.05). ** statistically significant difference from the G1W and G2W groups (*P* < 0.05). **C**, The average size of the periapical lesion of the distal root for each group. * statistically significant difference from the G1W group (*P* < 0.05). ** statistically significant difference from the G1W and G2W groups (*P* < 0.05).

Figure 2A shows the histopathologic changes in the pulpal and periradicular tissue as the apical periodontitis progressed. In the G1W group, pulp necrosis was observed below the exposure site, and neutrophils were densely packed under the necrosis site (Figure 2A5–7). Moreover, the proliferation of fibroblasts and blood vessels was observed in the living pulp space of the root canal (Figure S1A). In the G2W group, the pulp necrosis level extended down into the apical third of the root, and in some cases, the entire pulp was necrotized. Proliferated fibroblasts, highly populated neutrophils, and scattered lymphocytes were observed mostly in the periapical area. Some specimens showed densely packed neutrophils around the root apex. Multinucleated osteoclasts were gathered around the adjacent alveolar bone surface and demonstrated a resorptive appearance with remodeling (Figure 2A9–11 and Figure S1B). In the G3W group, a mixture of living neutrophils and dead neutrophil debris was observed around the root apex. The size of the periapical lesion increased as the fibrosis progressed and accelerated the resorption of alveolar bone as compared with the G1W and G2W groups (Figure 2A13–15 and Figure S1C). The mean size of the periradicular lesion of the distal root was estimated as 0.53 ± 0.12, 1.36 ± 0.34, and 3.35 ± 0.68 mm^2^ for the G1W, G2W, and G3W groups, respectively, indicating that the periapical lesion grew larger over time (Figure 2A4,8,12,16 and Figure 2C). These findings, taken together, show that pulp necrosis gradually progressed from the pulp chamber toward the root apex (Figure 2B), and periapical inflammation and alveolar bone destruction around the root apex were also exacerbated as the disease progressed over time.

### 3.3. Richness of Bacterial Species

**Figure 3:**
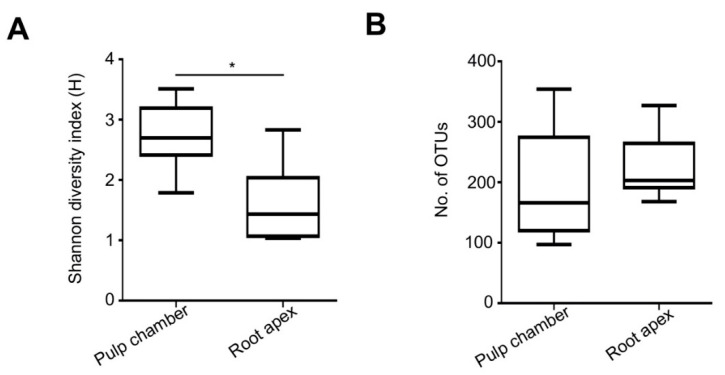
Diversity and species-level operational taxonomic units in the pulp chamber and the root apex. (**A**) Shannon diversity index in the pulp chamber and the root apex. Alpha diversity and Shannon diversity index are plotted for the pulp chamber and the root apex. The line inside the box indicates the median. * *P* < 0.05. (**B**) Species-level operational taxonomic units in the pulp chamber and the root apex. The cutoff value for assigning a sequence to a species-level phylotype was 97% similarity. Cluster_fast command was used to conduct de novo clustering to generate operational taxonomic units (OTUs).

To assess the correlation between the microbiota and apical periodontitis in rats, we used pyrosequencing to analyze the bacterial communities in the pulp chamber and root apex. We obtained a total 1,305,708 reads. A mean number of reads per sample of 72,539 ± 7454 was obtained. The results for each sample are summarized in Table 1. We used the EzTaxon-extended database for the taxonomic assignment of each pyrosequencing read. A total of 27 phyla, 645 genera, and 1276 species were detected. The number of species-level OTUs observed in the pulp chamber, and root apex were 195.2 and 223.6, respectively (*P* = 0.233; Figure 3A). Shannon diversity in the pulp chamber and root apex was 2.74 and 1.61, respectively (Figure 3B), indicating that the pulp chamber had a higher diversity of bacterial species than that of the root apex (*P* = 0.004). Unlike the pulp chamber (Shannon index [H]; 2.80 ± 0.533, 2.53 ± 0.657, and 2.89 ± 0.548 for the G1W, G2W, and G3W groups, respectively; Figure S2A), increased microbial diversity was found in the root apex during disease development (1.05 ± 0.032, 1.71 ± 0.432, and 2.08 ± 0.704 for the G1W, G2W, and G3W groups, respectively; Figure S2B). These results indicate that specific bacterial communities in the root apex might be closely associated with disease progression.

### 3.4. Comparison of Bacterial Communities

**Figure 4:**
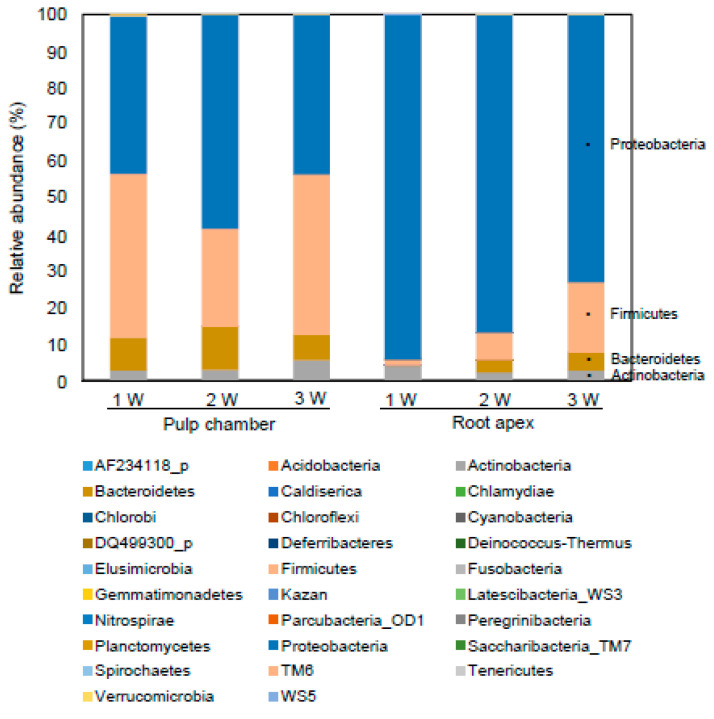
Relative abundance of bacterial phyla identified in the pulp chamber and the root apex. After removing the chimeric sequences, the EzTaxon-e database, which contains the bacterial 16S rRNA gene sequence of type strains, was used for the taxonomic assignment of each read. The relative abundance of bacteria phyla was analyzed in EzBioCloud 16S-based microbiome taxonomic profiling (MTP).

Next, to determine the chronological changes in the endodontic microbiota with the development of apical periodontitis, the bacterial community at the phylum and species levels was compared among the G1W, G2W, and G3W groups. Proteobacteria (43.11%, 58.61%, and 43.50% for the G1W, G2W, and G3W groups, respectively) and Firmicutes (44.69%, 26.75%, and 43.94% for the G1W, G2W, and G3W groups, respectively) were the dominant phyla in the pulp chamber (Figure 4 and Figure S3). Proteobacteria, the most dominant phylum in the root apex, decreased proportionally with disease progression (94.19%, 86.92%, and 73.16% for the G1W, G2W, and G3W groups, respectively). By contrast, the phylum Firmicutes increased proportionally with disease progression (1.61%, 7.47%, and 19.20% for the G1W, G2W, and G3W groups, respectively; Figure 4 and Figure S3). Thus, the phylum of the root apex showed a disease-dependent tendency compared with that of the pulp chamber.

**Figure 5:**
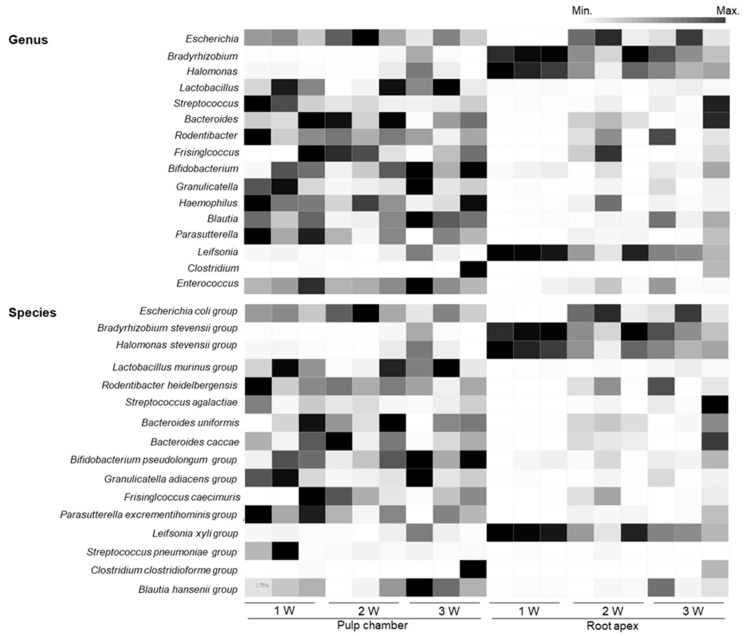
Heat map of the relative abundance of bacterial genera and species identified in the pulp chamber and the root apex. Any phylotype spanning more than 1% in at least one individual sample tested was included in the representative species. Colors reflect relative abundance from low (white) to high (black).

As shown in Figure 5, at the genus level, *Escherichia, Streptococcus, Lactobacillus, Rodentibacter*, and *Bacteroidetes* were the dominant genera in the pulp chamber. The most abundant and prevalent genera in the root apex were *Bradyrhizobium, Halomonas*, and *Escherichia*. Particularly, *Bradyrhizobium* accounted for 54.26% genera in the G1W group and decreased to 33.08% and 28.41% in the G2W and G3W groups, respectively. *Halomonas* also decreased as the disease progressed (39.26%, 17.76%, and 19.49% for the G1W, G2W, and G3W groups, respectively). The genus *Escherichia* was observed at high levels in the G2W (32.10%) and G3W (21.77%) groups but not in the G1W group (0%). At the species-level, *Escherichia coli* (23.70%, 43.27%, and 17.37% for the G1W, G2W, and G3W groups, respectively) was the most dominant species in the pulp chamber (Figure 5 and Figure S4). Moreover, the species *Azospirillum oryzae* in the pulp chamber increased as the disease progressed (0%, 0.001%, and 0.065% for the G1W, G2W, and G3W groups, respectively; *P* = 0.034). The species *Bradyrhizobium japonicum* (38.58%) and *Halomonas stevensii* (25.11%) were observed in high levels in the apical part. Particularly, *B. japonicum* group and *H. stevensii* group in the root apex decreased as the disease progressed. *E. coli* in the root apex accounted for 0.07% bacteria in the G1W group and increased to 32.10% and 21.77% in the G2W and G3W groups, respectively.

### 3.5. Pattern of Bacterial Community Based on Phylogenetic Analysis

**Figure 6:**
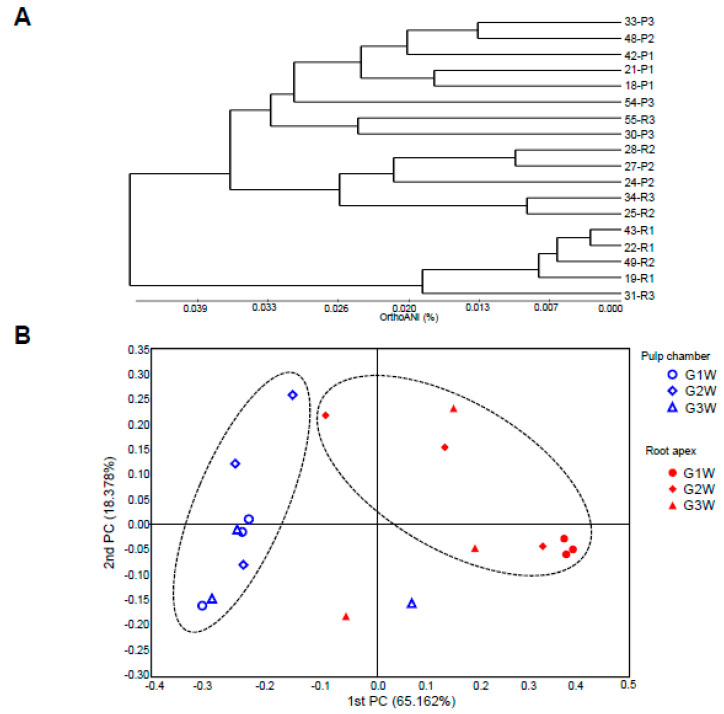
Principal coordinates analysis (PCoA) of the 18 bacterial communities at the species-level based on the microbiota. The beta-diversity distances were calculated using Fast UniFrac. (**A**) unweighted pair group method with arithmetic mean (UPGMA) dendrogram showing the relationship between 18 individual samples from the pulp chamber and root apex. (**B**) PCoA of UniFrac distances on 18 individual samples. Circles of the dashed lines indicate the cluster.

To determine the relationship of the bacterial communities between the pulp chamber and the root apex, we used weighted Fast UniFrac analysis to obtain phylogenetic trees (Figure 6A). This result indicates that the bacterial community of the root apex was distinct from that of the pulp chamber. In the UniFrac-based principal coordinates analysis, the first two principal components showed 83.53% variation, with a distinctive clustering of the two different groups (Figure 6B). These results suggest that specific bacterial populations are associated with two different habitats, namely, the pulp chamber and root apex. Moreover, the bacterial communities in the root apex were separated distinctly into two main clusters related to the G1W and G2W/G3W groups (Figure S2C), indicating that the bacterial communities in the root apex may represent a specific signature of microbial community shifts associated with disease progression.

## 4. Discussion

We established an experimental rat model of apical periodontitis to investigate the relationship between the pathogenesis of endodontic infection and microbial communities during disease progression. Indeed, the pulp exposure of the rat molar led to pulpal necrosis and periapical lesion, resulting in alveolar bone destruction and periapical abscess formation over time, which is the typical progression of apical periodontitis [3]. Although rat apical periodontitis models to investigate endodontic infection have been introduced, most studies have focused on periapical lesions and histologic changes at a specific time point [14,22], and the chronological changes in the microbiota together with radiologic and histologic analyses has been poorly studied. Notably, there are some previous reports available on the induction of murine apical periodontitis by *Enterococcus faecalis* or *Fusobacterium nucleatum* [23,24] and rat chronic apical periodontitis by *Porphyromonas gingivalis* or *E. faecalis* [25]. Nonetheless, those reports have seemingly only limited applicability for the comprehensive understanding of the role of microbial communities responsible for the disease progress. To the best of our knowledge, this is the first study on the chronological changes of microbiota combined with radiologic and histologic analyses.

In the radiologic and histologic analyses of this study, we found that simple pulp exposure could lead to rapid progress of pulpal necrosis, immune cell infiltration throughout the pulp and periapical lesion, and alveolar bone resorption in the periradicular area through three weeks, which is consistent with previous studies [3,26,27,28]. Representative studies on rat models of apical periodontitis reported that periapical lesions actively expanded for 3–4 weeks after pulp exposure (active phase) and showed slower progress thereafter (chronic phase) [14,26,29]. The linear increase in alveolar bone destruction around the root apices in this study implies that our observation period is within the active phase of apical periodontitis. In particular, the G2W group appears to have shown a sharp increase in bone destruction compared with the G1W group in micro-CT analysis. The magnified view of periapical lesion also showed frequent presence of osteoclasts around the resorbed alveolar bone in the G2W group; 1–2 weeks is assumed to be a period of markedly rapid inflammatory change within the active phase of apical periodontitis based on the progress of pulp necrosis level and alveolar bone resorption.

Proteobacteria, Firmicutes, Bacteroidetes, and Actinobacteria were dominant phyla in both the pulp chamber and root apex of the rat apical periodontitis model. All of these phyla were also reported to be present in rat tongues despite a difference in Actinobacteria: 5% of microbial phyla in the rat pulp and root apex versus 40–60% of microbial phyla in rat tongues [30]. Interestingly, most of those phyla were also dominant in the infected root apex of humans [31,32,33]. However, there were some differences: Fusobacteria was not found in the present study but was a dominant phylum in the human infected root. Moreover, reduced Proteobacteria with elevated Firmicutes were observed in the roots of rats, whereas both of these phyla were reduced in human infected roots [34]. Conversely, the genera *Streptococcus*, *Haemophilus*, *Actinomyces*, *Granulicatella*, and *Leptotrichia* were reported to decrease in the human samples of apical periodontitis compared with those of healthy subjects [34]. Among these, in the present study, only Streptococcus was decreased in the pulp chamber during the progression of apical periodontitis.

The microbial signature of the rat root apex appears to better represent apical periodontitis than that of the pulp chamber, because the microbiota of root apex tends to gradually change over time as the disease progresses. Unlike the microbial composition in the pulp chamber, *H. stevensii* and *B. japonicum* were the predominant species in the G1W group, and *B. japonicum* and *E. coli* were most prevalent in the root apex in the G2W and G3W groups. Reduced levels of *H. stevensii* during the development of apical periodontitis were observed. Indeed, one study reported that the microbes present in the root are involved directly in the pathological changes of the apical lesion [35]. Because of the vicinity of exposed dental pulp to the oral environment, one may think that saliva microbes may be easily mixed with and thereby influence the microbes of the pulp chamber. Thus, we further analyzed the composition of the microbiota in rat saliva. Interestingly, the microbial composition of saliva was completely different from that of the pulp chamber, but it was rather similar to that of the root apex (Figure S5A–C). This may be because (1) microbes in the oral cavity are continuously washed away by saliva, (2) the microenvironment of the pulp chamber is influenced by various cells and factors in the unique pulp condition, and (3) the pulp chamber is isolated enough to form its own particular microbiota. Therefore, changes in the microbial communities in the root apex may be closely related to the pathogenesis of apical periodontitis.

In this study, we demonstrated an experimental platform that can be used to study apical periodontitis in detail with induction of pulpal necrosis and periapical lesions. Using this rat apical periodontitis model, we demonstrated the chronological changes in the microbiota during the apical periodontitis progression. Therefore, the use of a rat model of apical periodontitis alongside an analysis of the endodontic microbiota profile concerning histopathology would contribute to a better understanding of the apical periodontitis pathogenesis and could be used as a platform for the development of new treatment methods.

## Figures and Tables

**Table 1:** Summary of pyrosequencing.

	Total Valid Reads	Average Read Lengths	No. of OTUs	Shannon Diversity Index	No. of Phylum	No. of Genus	No. of Species
42-P1	65507	419.3 bp	212	3.39	5	113	184
21-P1	72343	425.2 bp	99	2.35	5	61	95
18-P1	70389	424.9 bp	97	2.66	5	51	84
48-P2	65178	422.1 bp	194	2.99	6	109	180
27-P2	71754	423.9 bp	166	1.78	4	91	141
24-P2	66468	420.2 bp	354	2.83	5	149	328
54-P3	64866	415.1 bp	337	3.51	6	136	310
33-P3	67652	421.7 bp	157	2.69	5	95	145
30-P3	89946	418.3 bp	141	2.47	9	88	137
43-R1	74667	410.7 bp	199	1.09	15	138	196
22-R1	76408	411.5 bp	192	1.03	13	139	186
19-R1	67556	412.9 bp	168	1.04	14	131	166
49-R2	64582	410.7 bp	203	1.23	12	151	202
28-R2	84751	421.0 bp	205	2.07	11	117	185
25-R2	79475	418.2 bp	327	1.83	14	205	319
55-R3	76542	417.5 bp	294	2.83	9	176	284
34-R3	68434	418.9 bp	235	1.43	15	164	229
31-R3	80190	414.3 bp	190	2.00	13	135	188

## Supplementary Materials

The following are available online at https://www.mdpi.com/2076-2607/8/8/1174/s1, Figure S1. Histopathologic features of the G1W, G2W, and G3W groups; Figure S2. Diversity and species-level operational taxonomic units in the root apex; Figure S3. Relative abundance of bacterial phyla in the individual samples from the pulp chamber and the root apex; Figure S4. Relative abundance of bacterial species identified in the individual samples from the pulp chamber and the root apex; Figure S5: Pyrosequencing analysis of rat salivary microbiota.

## References

[1] Ricucci D., Siqueira J.F. (2010). Biofilms and apical periodontitis: Study of prevalence and association with clinical and histopathologic findings. J. Endod..

[2] Kirkevang L.L., Vaeth M., Horsted-Bindslev P., Bahrami G., Wenzel A. (2007). Risk factors for developing apical periodontitis in a general population. Int. Endod. J..

[3] Nair P.N. (2004). Pathogenesis of apical periodontitis and the causes of endodontic failures. Crit. Rev. Oral Biol. Med..

[4] Le Goff A., Bunetel L., Mouton C., Bonnaure-Mallet M. (1997). Evaluation of root canal bacteria and their antimicrobial susceptibility in teeth with necrotic pulp. Oral Microbiol. Immunol..

[5] Siqueira J.F., Rocas I.N. (2005). Exploiting molecular methods to explore endodontic infections: Part 2--Redefining the endodontic microbiota. J. Endod..

[6] Sundqvist G. (1992). Associations between microbial species in dental root canal infections. Oral Microbiol. Immunol..

[7] Siqueira J.F., Rocas I.N. (2009). Community as the unit of pathogenicity: An emerging concept as to the microbial pathogenesis of apical periodontitis. Oral Surg. Oral Med. Oral Pathol. Oral Radiol. Endod..

[8] Ozok A.R., Persoon I.F., Huse S.M., Keijser B.J., Wesselink P.R., Crielaard W., Zaura E. (2012). Ecology of the microbiome of the infected root canal system: A comparison between apical and coronal root segments. Int. Endod. J..

[9] Shin J.M., Luo T., Lee K.H., Guerreiro D., Botero T.M., McDonald N.J., Rickard A.H. (2018). Deciphering Endodontic Microbial Communities by Next-generation Sequencing. J. Endod..

[10] Santos A.L., Siqueira J.F., Rocas I.N., Jesus E.C., Rosado A.S., Tiedje J.M. (2011). Comparing the bacterial diversity of acute and chronic dental root canal infections. PLoS ONE.

[11] Tzanetakis G.N., Azcarate-Peril M.A., Zachaki S., Panopoulos P., Kontakiotis E.G., Madianos P.N., Divaris K. (2015). Comparison of Bacterial Community Composition of Primary and Persistent Endodontic Infections Using Pyrosequencing. J. Endod..

[12] Anderson A.C., Al-Ahmad A., Elamin F., Jonas D., Mirghani Y., Schilhabel M., Karygianni L., Hellwig E., Rehman A. (2013). Comparison of the bacterial composition and structure in symptomatic and asymptomatic endodontic infections associated with root-filled teeth using pyrosequencing. PLoS ONE.

[13] Clemente J.C., Ursell L.K., Parfrey L.W., Knight R. (2012). The impact of the gut microbiota on human health: An integrative view. Cell.

[14] Stashenko P., Wang C.Y., Tani-Ishii N., Yu S.M. (1994). Pathogenesis of induced rat periapical lesions. Oral Surg. Oral Med. Oral Pathol..

[15] Halheit S., Benabdelkader S. Rigid Image Registration using Mutual Informationand Wavelet Transform. Proceedings of the International Conference on Signal, Image, Vision and their Applications (SIVA).

[16] Khanmohammadia S., Adibeig N., Shanehbandy S. (2017). An improved overlapping k-means clustering method for medical applications. Expert Syst. Appl..

[17] Hore S., Chakraborty S., Chatterjee S., Dey N., Ashour A.S., Chung L.V., Le D.-N. (2016). An Integrated Interactive Technique for Image Segmentation using Stack based Seeded Region Growing and Thresholding. Int. J. Electr. Comput. Eng..

[18] An S.M., Kim M.J., Seong K.Y., Jeong J.S., Kang H.G., Kim S.Y., Kim D.S., Kang D.H., Yang S.Y., An B.S. (2019). Intracutaneous Delivery of Gelatins Reduces Fat Accumulation in Subcutaneous Adipose Tissue. Toxicol. Res..

[19] Silva R.A.B., Sousa-Pereira A.P., Lucisano M.P., Romualdo P.C., Paula-Silva F.W.G., Consolaro A., Silva L.A.B., Nelson-Filho P. (2020). Alendronate inhibits osteocyte apoptosis and inflammation via IL-6, inhibiting bone resorption in periapical lesions of ovariectomized rats. Int. Endod. J..

[20] Hur M., Kim Y., Song H.R., Kim J.M., Choi Y.I., Yi H. (2011). Effect of genetically modified poplars on soil microbial communities during the phytoremediation of waste mine tailings. Appl. Environ. Microbiol..

[21] Park O.J., Yi H., Jeon J.H., Kang S.S., Koo K.T., Kum K.Y., Chun J., Yun C.H., Han S.H. (2015). Pyrosequencing Analysis of Subgingival Microbiota in Distinct Periodontal Conditions. J. Dent. Res..

[22] Yamasaki M., Kumazawa M., Kohsaka T., Nakamura H., Kameyama Y. (1994). Pulpal and periapical tissue reactions after experimental pulpal exposure in rats. J. Endod..

[23] Santa-Rosa C.C., Thebit M.M., Maciel K.F., Brito L.C.N., Vieira L.Q., Ribeiro-Sobrinho A.P. (2018). Evaluation of chemokines and receptors in gnotobiotic root canal infection by F. nucleatum and E. faecalis. Braz. Oral Res..

[24] Wu Y., Sun H., Yang B., Liu X., Wang J. (2018). 5-Lipoxygenase Knockout Aggravated Apical Periodontitis in a Murine Model. J. Dent. Res..

[25] Chen S., Lei H., Luo Y., Jiang S., Zhang M., Lv H., Cai Z., Huang X. (2019). Micro-CT analysis of chronic apical periodontitis induced by several specific pathogens. Int. Endod. J..

[26] Kakehashi S., Stanley H.R., Fitzgerald R.J. (1965). The Effects of Surgical Exposures of Dental Pulps in Germ-Free and Conventional Laboratory Rats. Oral Surg. Oral Med. Oral Pathol..

[27] Lin S.K., Hong C.Y., Chang H.H., Chiang C.P., Chen C.S., Jeng J.H., Kuo M.Y. (2000). Immunolocalization of macrophages and transforming growth factor-beta 1 in induced rat periapical lesions. J. Endod..

[28] Anan H., Akamine A., Hara Y., Maeda K., Hashiguchi I., Aono M. (1991). A histochemical study of bone remodeling during experimental apical periodontitis in rats. J. Endod..

[29] Akamine A., Anan H., Hamachi T., Maeda K. (1994). A histochemical study of the behavior of macrophages during experimental apical periodontitis in rats. J. Endod..

[30] Hyde E.R., Luk B., Cron S., Kusic L., McCue T., Bauch T., Kaplan H., Tribble G., Petrosino J.F., Bryan N.S. (2014). Characterization of the rat oral microbiome and the effects of dietary nitrate. Free Radic. Biol. Med..

[31] Keskin C., Demiryurek E.O., Onuk E.E. (2017). Pyrosequencing Analysis of Cryogenically Ground Samples from Primary and Secondary/Persistent Endodontic Infections. J. Endod..

[32] Siqueira J.F., Alves F.R., Rocas I.N. (2011). Pyrosequencing analysis of the apical root canal microbiota. J. Endod..

[33] Siqueira J.F., Antunes H.S., Rocas I.N., Rachid C.T., Alves F.R. (2016). Microbiome in the Apical Root Canal System of Teeth with Post-Treatment Apical Periodontitis. PLoS ONE.

[34] Qian W., Ma T., Ye M., Li Z., Liu Y., Hao P. (2019). Microbiota in the apical root canal system of tooth with apical periodontitis. BMC Genom..

[35] Gomes B., Herrera D.R. (2018). Etiologic role of root canal infection in apical periodontitis and its relationship with clinical symptomatology. Braz. Oral Res..

