# Supplementary material for: A Pilot Study of Chronological Microbiota Changes in a Rat Apical Periodontitis Model
Source: Microorganisms. 2020 Aug 2;8(8):1174. doi: 10.3390/microorganisms8081174 (PMC7464309; doi:10.3390/microorganisms8081174)
Supplement: Supplementary file 1 [file microorganisms-08-01174-s001.pdf]

microorganisms

Chronological microbiota changes in rat apical periodontitis model

Ok-Jin Park, Moon-Hee Jung, Eun-Hye Lee, Mi-Ran Cho, Jae-Hong Hwang, Seung-Ryong Cho, Cheol-Heui Yun, Seung Hyun Han, Sun-Young Kim

Supplementary Figure and Figure legends

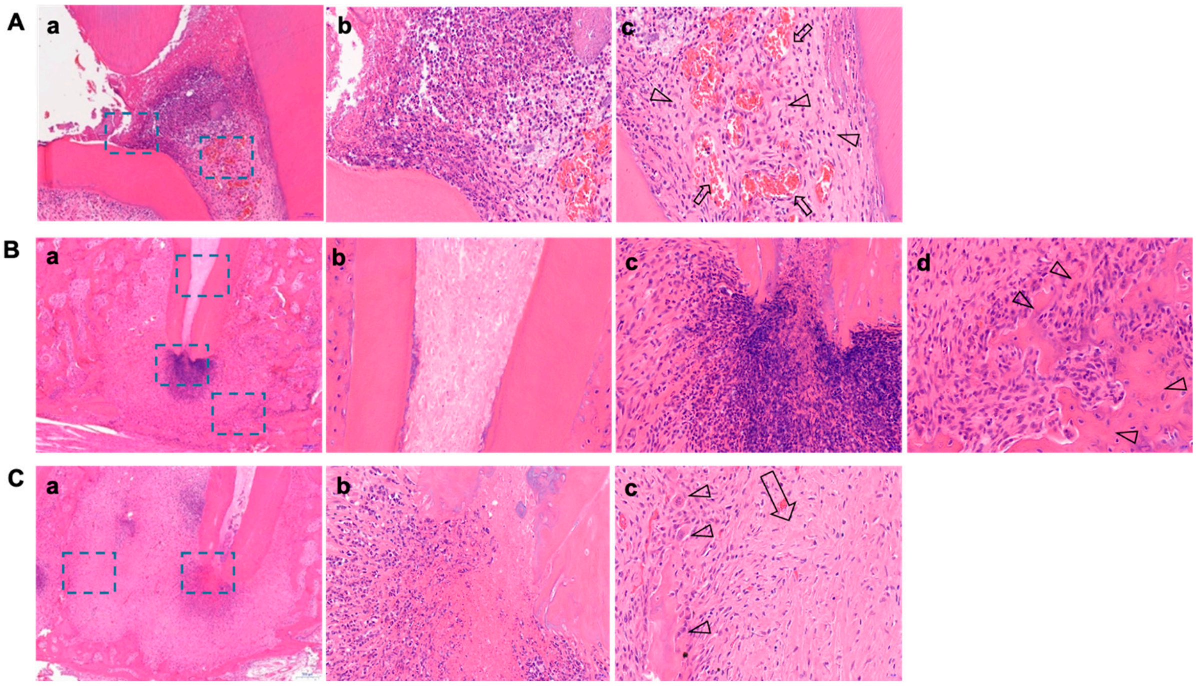

Figure S1. Representative magnified histologic features of the chronological change in the pulp-exposed lower first molar of rat. (**A**) Histopathologic features of G1W. (a) Coronal pulp below the exposure site. (b) Magnified view of the necrosis site. Necrotic debris and many neutrophils were densely packed around the necrosis site. (c) Blood vessels (empty arrow) and fibroblasts (empty arrowhead) were proliferated in the living pulp space below the necrosis site. (**B**) Histopathologic features of G2W. (a) Apical area showing whole pulp necrosis, apical inflammation, and alveolar bone resorption. (b) Magnified apical one-third of the root canal. Cell morphology is faint, and nuclei are barely visible because of progressing necrosis. (c) Magnified view of the apical area. Densely packed neutrophils and scattered lymphocytes were observed around the root apex. (d) Alveolar bone surface was uneven, and osteoclasts (empty arrowhead) were attached to the surface. (**C**)

Histopathologic features of G3W. (a) Apical area that showed extensive inflammation and alveolar bone resorption. (b) Magnified view of the root apical area. Alive and dead neutrophils and necrotic debris were mixed around the apex. (c) Extensive alveolar bone resorption and progressed fibrosis (empty arrow) were observed where the alveolar bone resorbed. Osteoclasts (empty arrowhead) were attached around the remaining alveolar bone surface.

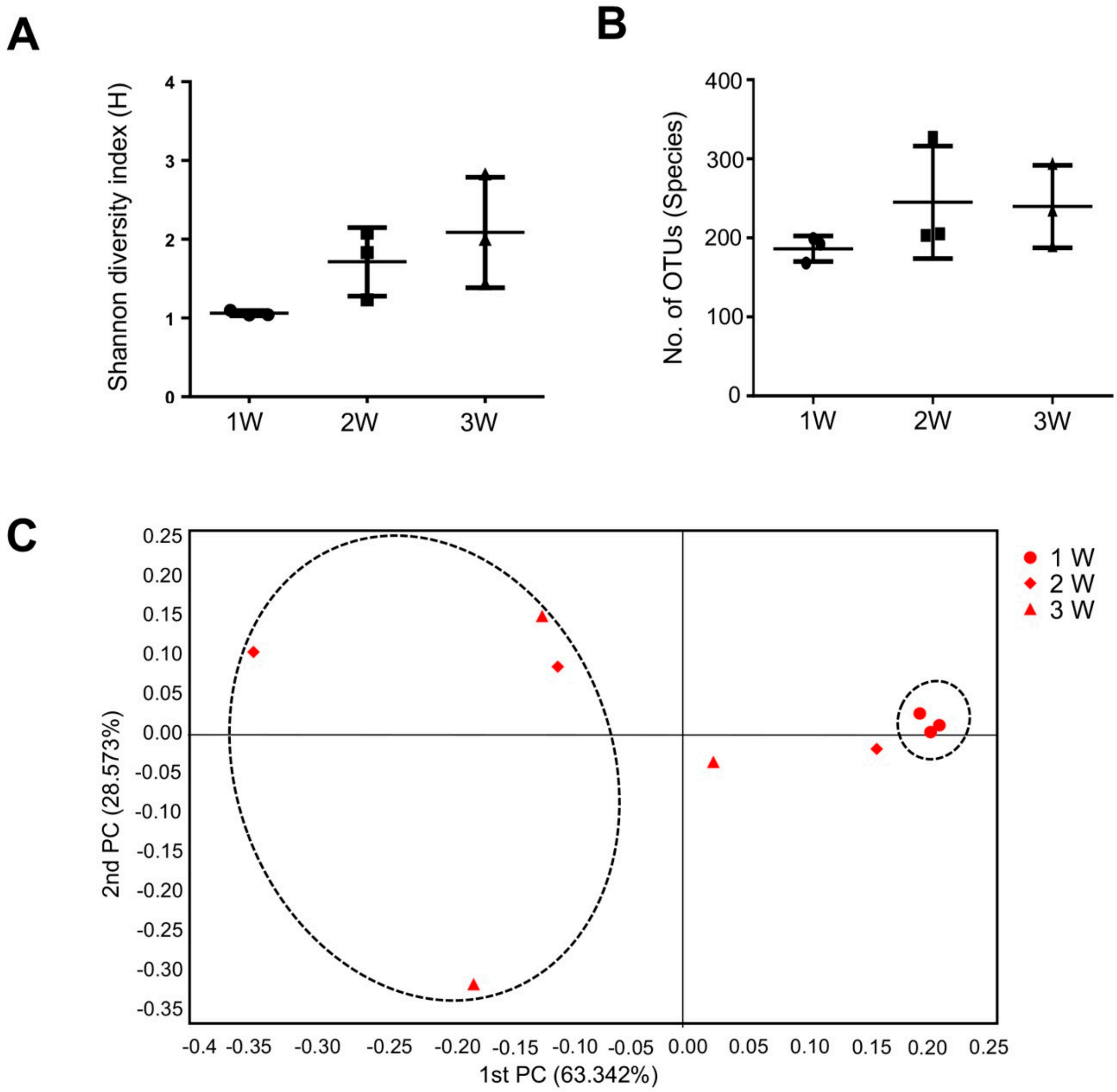

Figure S2. Diversity and species-level operational taxonomic units in the root apex. (**A**) Shannon diversity index in the root apex. (**B**) Number of operational taxonomic units in the root apex. (**C**) Principal coordinates analysis (PCoA) of the bacterial communities in the root apex displaying two main clusters related to the G1W and G2W/G3W groups.

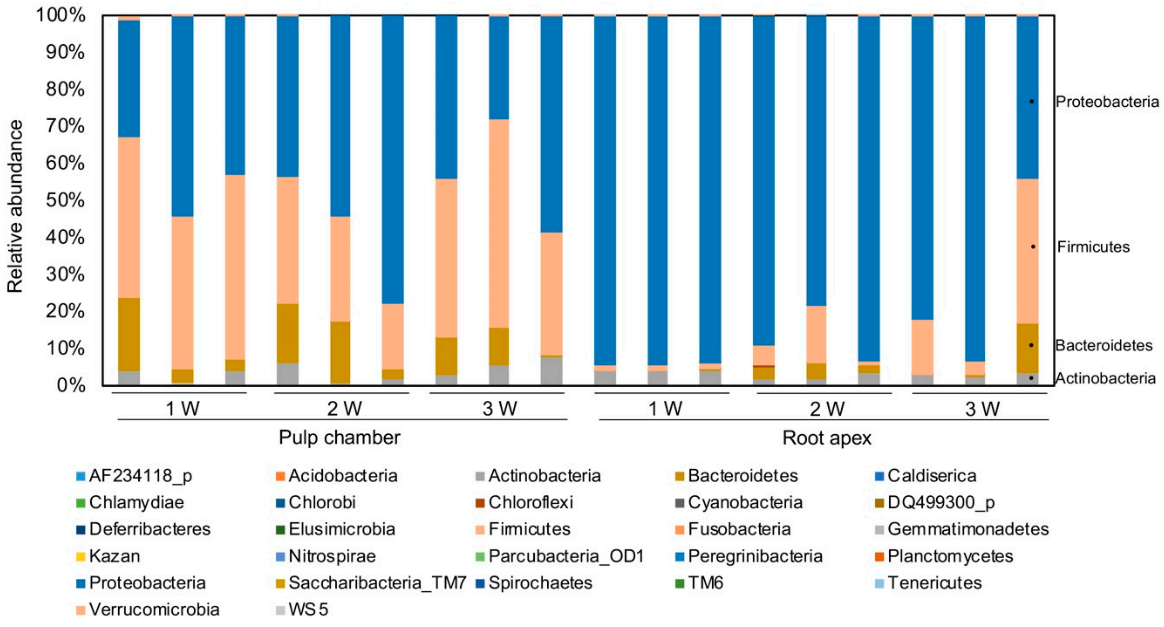

Figure S3. Relative abundance of bacterial phyla in the individual samples from the pulp chamber and the root apex.

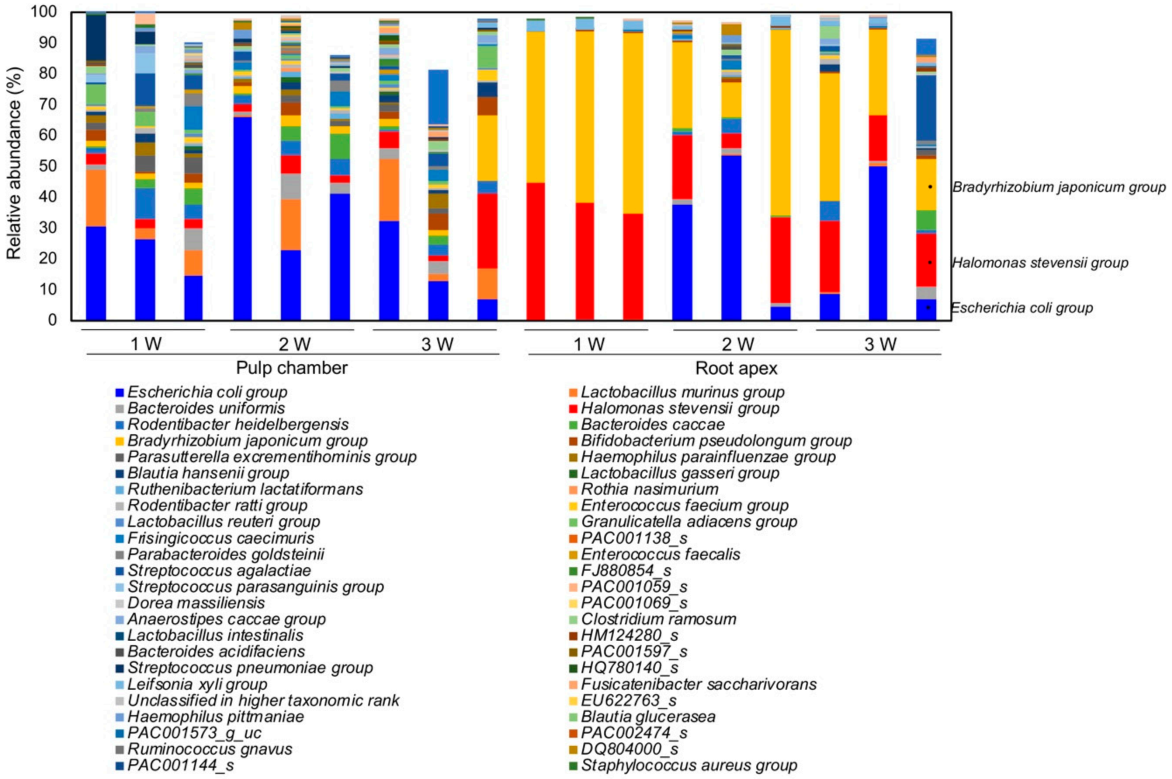

Figure S4. Relative abundance of bacterial species identified in the individual samples from the pulp chamber and the root apex. The species occupying more than 1% in at least one individual sample are shown.

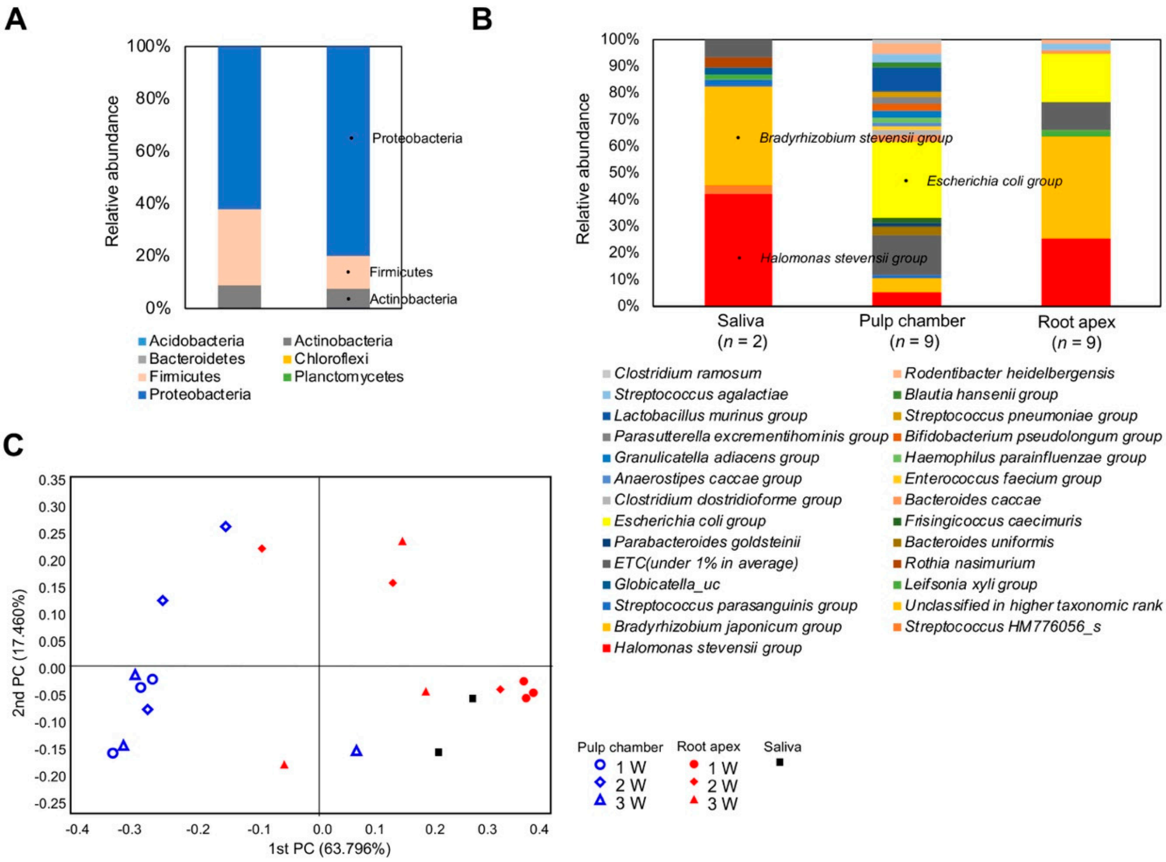

Figure S5. Pyrosequencing analysis of rat salivary microbiota. (**A**) Relative abundance of bacterial phyla identified in the saliva. (**B**) Relative abundance of species identified in the saliva, pulp chamber, and root apex. (**C**) Principal coordinates analysis (PCoA) of UniFrac distances on 20 individual samples.
